# The Sequence Characteristics and Expression Models Reveal Superoxide Dismutase Involved in Cold Response and Fruiting Body Development in *Volvariella volvacea*

**DOI:** 10.3390/ijms17010034

**Published:** 2015-12-29

**Authors:** Jun-Jie Yan, Lei Zhang, Rui-Qing Wang, Bin Xie, Xiao Li, Ren-Liang Chen, Li-Xian Guo, Bao-Gui Xie

**Affiliations:** 1 Mycological Research Center, College of Life Sciences, Fujian Agriculture and Forestry University, Fuzhou 350002, China; junjie017@163.com (J.-J.Y.); zhanglei311540@163.com (L.Z.); 13405987274@163.com (R.-Q.W.); 18705043595@163.com (B.X.); 18259088174@163.com (X.L.); CRLmiracle@126.com (R.-L.C.); LXG20150331@163.com (L.-X.G.); 2 College of Food Sciences, Fujian Agriculture and Forestry University, Fuzhou 350002, China

**Keywords:** straw mushroom, alternative splicing modes, gene differential expression, cold stress, fruiting-body development

## Abstract

As the first defence for cells to counteract the toxicity of active oxygen, superoxide dismutase (SOD) plays an important role in the response of living organisms to stress and cell differentiation. One extracellular Cu-ZnSOD (ecCu-ZnSOD), and two MnSODs, were identified based on the *Volvariella volvacea* genome sequence. All three genes have complicated alternative splicing modes during transcription; only when the fourth intron is retained can the *Vv_Cu-Znsod1* gene be translated into a protein sequence with SOD functional domains. The expression levels of the three *sod* genes in the pilei are higher than in the stipe. The *Vv_Cu-Znsod1* and the *Vv_Mnsod2* are co-expressed in different developmental stages of the fruiting body, with the highest level of expression in the pilei of the egg stage, and they show a significant, positive correlation with the efficiency of karyogamy, indicating the potential role of these two genes during karyogamy. The expression of the *ecCu-Znsod* and two *Vv_Mnsod* genes showed a significant up-regulated when treated by cold stress for one hour; however, the lack of the intracellular Cu-ZnSOD encoding gene (*icCu-Znsod*) and the special locus of the *ecCu-Znsod* gene initiation codon suggested a possible reason for the autolysis phenomenon of *V. volvacea* in cold conditions.

## 1. Introduction

Superoxide dismutases (SOD, EC 1.15.1.1) constitute a family of important enzymes that scavenge oxygen free radicals. As the first defence in cells to counteract the toxicity of active oxygen, these enzymes exist in various aerobic organisms [1,2]. SODs are classified into four groups according to their binding metal ions: Fe, Ni, Mn, and Cu-Zn. MnSOD and Cu-ZnSOD are found in eukaryotes and in most prokaryotes. FeSOD, however, is found mostly in prokaryotes and in a few plants, and NiSOD is only found in Streptomyces and cyanobacteria [3,4,5]. Many studies have indicated that although Cu-ZnSOD and MnSOD have different crystal structures, metal cofactors, catalytic mechanisms and amino acid sequences, they play similar and important roles in biological processes, including scavenging of reactive oxygen species (ROS), environmental stress responses, cell differentiation and ageing [6,7,8,9,10,11,12,13].

*Volvariella volvacea* is an important edible mushroom cultivated seasonally in tropical and subtropical areas, and the growth of the fruiting body is susceptible to environmental stress [14,15,16]. It is very sensitive to cold conditions that cause deterioration or even autolysis of hyphae, and have a negative impact on mushroom production and preservation [17,18]. Li *et al.* [19] determined the SOD enzyme activity and developed isozyme electrophoretograms for the different developmental stages of the fruiting body of *V. volvacea*. They found that the SOD enzyme activity increases gradually as the fruiting body matures, and the number of isozyme bands is the smallest when the activity of the enzyme is the highest. Li *et al.* [20] also found differences among SOD isozyme patterns in *V. volvacea* mycelia at different ages, in different growth periods of the fruiting body and in the fruiting body stored at a low temperature for different time periods. All of these results show that SOD is involved in regulating biological processes, including mycelial aging, fruiting body development and response to cold stress. Although, the *sod* genes of several mushrooms were studied [21,22], there has been no report of the identification of SOD encoding genes or of gene regulation at the transcriptional level in *V. volvacea*. Under laboratory conditions, we sequenced the genome and transcriptome of *V. volvacea* and identified genes encoding SOD using a bioinformatics annotation method. By analyzing the *sod* genetic structure and detecting its expression in different samples, we initiated studies of the biological function of SOD at the gene level and at the transcriptional level in *V. volvacea*.

## 2. Results

### 2.1. Annotation and Sequence Accuracy Verification of SOD Encoding Genes

The protein-protein BLAST (BLASTP) results showed that the *V. volvacea* genome contained one Cu-ZnSOD (encoding gene prediction No. GME1978) and two MnSODs (encoding gene prediction Nos. GME5987 and GME8899), but no FeSOD or NiSOD.

The DNA sequences encoding the three SODs were obtained from the genome based on the gene prediction numbers and gene models were annotated as described in the supplementary methods (S1). The results suggested that the lengths of GME1978 (*Vv_Cu-Znsod1*), GME5987 (*Vv-Mnsod1*), and GME8899 (*Vv-Mnsod2*) were 1255 bps, 1110 bps, and 1589 bps, respectively. The elongated sequences were mapped by the reads obtained by genome sequencing, and the results are shown in supplementary Figure S1. The base pairs of the full-length gene sequences had at least nine reads mapped. The sequencing results using the Sanger method confirmed that the three *sod* gene sequences were correct and could be used for further study.

### 2.2. Genetic Structure and Alternative Splicing Analysis

**Figure 1:**
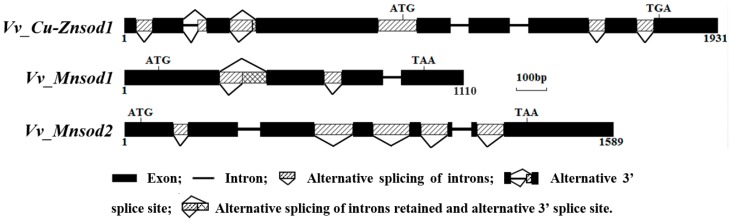
Schematic representation of the *V. volvacea sod* gene structures.

The result of remapping the transcriptome reads indicated that all three *sod* genes possessed introns, and several intron regions were alternatively spliced (see Supplementary Figure M-6 to Figure M-8). The results are shown in Figure 1, and the sequences are shown in supplementary sequences S1 to S3. All the verified nucleotide sequences from this study have been submitted to GenBank under the accession ID numbers KP747453, KP747454, and KP747455.

The *Vv_Cu-Znsod1* possessed eight introns, I1-I8, with lengths of 52, 76, 80, 125, 57, 58, 54, and 55 bp. Among them, intron retention existed for I1, I4, I7, and I8; I2 contained an alternative 3’ splice site, and I3 showed either intron retention or alternative 3’ splicing. The *Vv_Mnsod1* contained three introns (I1-I3) of 154, 57 and 56 bp, among which I1 and I2 had alternative splicing modes for intron retention, and I1 also had the 5’ alternative splice site. The *Vv_Mnsod2* contained seven introns. The sizes of I1–I7 were 47, 72, 126, 119, 88, 61, and 87, among which I1, I3, I4, I5, and I7 had alternative splicing sites for intron retention.

### 2.3. Alignment and Phylogenetic Analysis of Amino Acid Sequences

**Figure 2:**
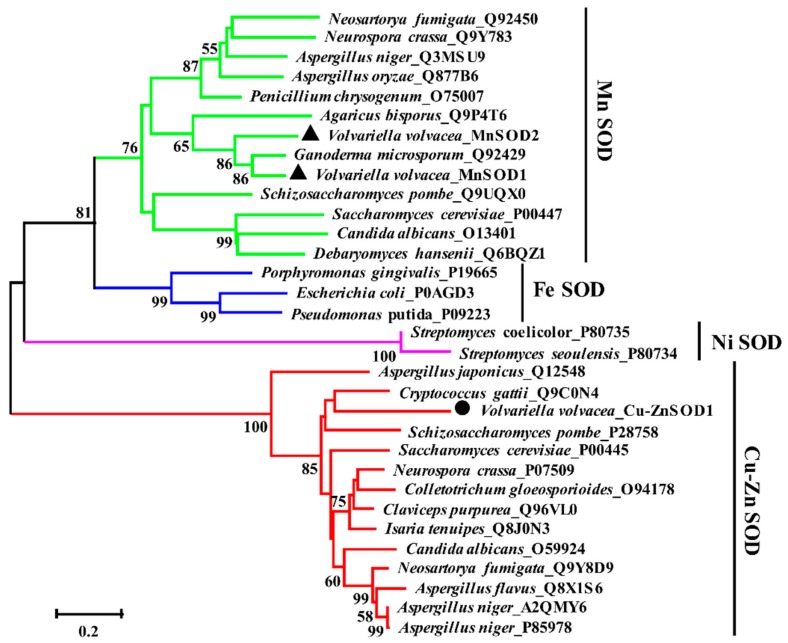
Neighbor-joining phylogenetic tree of SOD amino acid sequences across multiple species. Using the MUSCLE method to do the sequence alignment, the tree build was performed using the program MEGA version 5.1 and the “Partial Deletion” option to exclude positions covering less than 70% of the site. All of the sequences were downloaded from the UniProt database (http://www.uniprot.org/) except for three *V. volvacea* SODs. (The scale bar indicates evolutionary distance. Values above the branches indicate the degree of bootstrap support from a 1000 replicate analysis. One Cu-ZnSOD and two MnSODs of *V. volvacea* were labeled by black circular and triangle, respectively.)

As shown in Figure 2, 35 microorganism SOD sequences can be gathered into four branches of Cu-ZnSOD, MnSOD, FeSOD, and NiSOD by the Neighbor-joining (NJ) phylogenetic tree. Among them, *Volvariella volvacea*_MnSOD1, *Volvariella volvacea*_MnSOD2, and *Ganoderma microsporum*_Q92429 were on the same branch, and the branch support degree was up to 86%. The *Volvariella volvacea*_Cu-ZnSOD1 and the Cu-ZnSODs of other fungi were on the same branch, with 85% of the support degree. The tree revealed that, although attached to the Cu-ZnSOD branch, the *Aspergillus japonicus*_Q12548 was markedly different from the Cu-ZnSOD sequences of other fungi.

**Figure 3:**
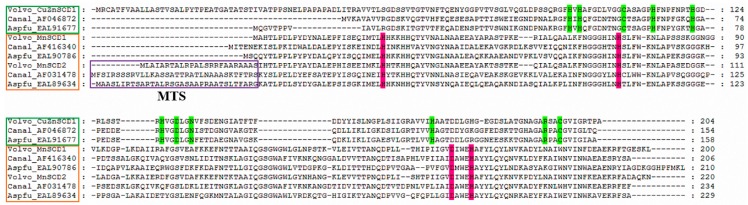
Multiple alignment of SOD amino acid sequences from *V. volvacea* (Volvo), *A. fumigatus* (Aspfu) and *C. albicans* (Canal). The sequences were downloaded from the NCBI database (http://www.ncbi.nlm.nih.gov/protein/) except for the *V. volvacea* SODs. The name (ID number) of Cu-ZnSOD and MnSOD sequences were marked by the green and yellow boxes, respectively. The mitochondrial targeting sequence (MTS) is boxed in purple; the metal binding sites (H97, H99, H114 and H171 for Cu^2+^; H114, H122, H131, and D134 for Zn^2+^), C108, and C147 involved in formation of a disulﬁde bond and N137, which acts as a potential glycosylation site, are highlighted with green [11,12]. The potential metal-binding sites for Mn^2+^ are highlighted in pink [13].

Based on the findings of several studies [23,24], the Cu-ZnSOD and the MnSOD amino acid sequences of *Aspergillus fumigatus* and *Candida albicans* were downloaded from the NCBI database, and the multiple sequence alignment of the three SOD amino acid sequences of the *V. volvacea* was performed using the multiple sequence comparison by log-expectation (MUSCLE) method. As shown in Figure 3, the results indicated that all three SOD amino acid sequences of *V. volvacea* possess conserved amino acid residues involved in metal ion binding. The N terminus of the Vv_MnSOD2 contains a 24 amino acid residues mitochondrial targeting sequence (MTS), which is not detected in the Vv_MnSOD1. This observation indicates that the two MnSODs in *V. volvacea* play roles in the mitochondria and cytoplasm, respectively, which is consistent with the prediction of their subcellular localization. According to its subcellular localization, the Vv_Cu-ZnSOD1 participates in the secretory pathway. The prediction of the signal peptide also indicated that the Vv_Cu-ZnSOD1 has a 17 amino acid residues signal peptide sequence belonging to a secretory protein, which indicated that the *Vv_Cu-Znsod1* gene encoding an extracellular Cu-ZnSOD protein (supplementary Figure S2).

### 2.4. Differential Expression of Sod Genes under Short-Term Cold Stress

**Figure 4:**
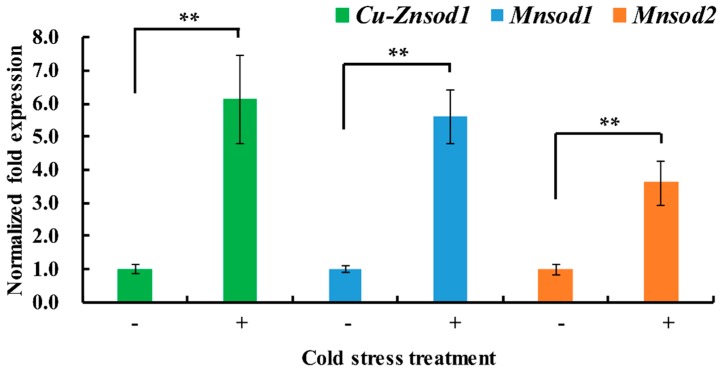
Differential expression of *sod* genes in *V. volvacea* H1521 stressed at low temperature for different times. Control (−), hyphae were incubated in phosphate buffered solution (PBS) buffer (0.02 mol/L, pH 8, 34 °C) for 1.5 h at 34 °C. Cold stress treated (+), hyphae were incubated in PBS buffer (0.02 mol/L, pH 8, 34 °C) for 0.5 h at 34 °C and then in PBS buffer (0.02 mol/L, pH 8, 4 °C) at 4 °C for 1 h. Data are the averages of four independent experiments. The error bars show the standard error of the mean. Statistical analysis was performed using least significant difference (LSD) test, ** *p* < 0.01.

To test the transcriptional responses of three *Vv-sod* genes to cold stress, the *V. volvacea* colonies submerged in phosphate buffer were switched from an incubation temperature of 34 to 4 °C. After 1 h of cold stress, transcript levels of *Vv_Cu-Znsod1*, *Vv_Mnsod1*, *and Vv_Mnsod2* in the cold-treated mycelia were increased by 5.1-fold, 4.6-fold, and 2.6-fold, respectively (Figure 4).

### 2.5. Expression Patterns of Sod Genes during the Fruiting-body Development Stages of V. volvacea

**Figure 5:**
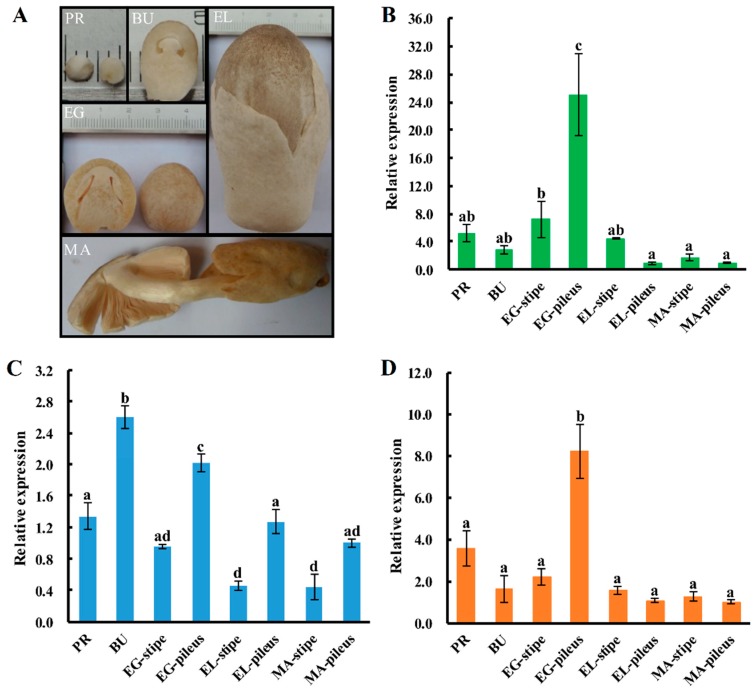
Expression patterns of *sod* genes in different tissues during different developmental stages of *V. volvacea* H1521. (**A**) the different fruiting body growth stages of *V. volvacea*; (**B**) the differential expression pattern of *Cu-Znsod1* gene; (**C**) the differential expression pattern of *Mnsod1* gene; and (**D**) the differential expression pattern of *Mnsod2* gene. PR = primordia; BU = button stage; EG = egg stage; EL = elongation stage; MA = maturation stage. The gene expression levels are presented relative to that in the MA-pileus. The means of three independent experiments are shown. The error bars show the standard error of the mean, and the different letters over the columns within a graph denote significant differences (*p* < 0.05, LSD test, *n* = 3).

**Figure 6:**
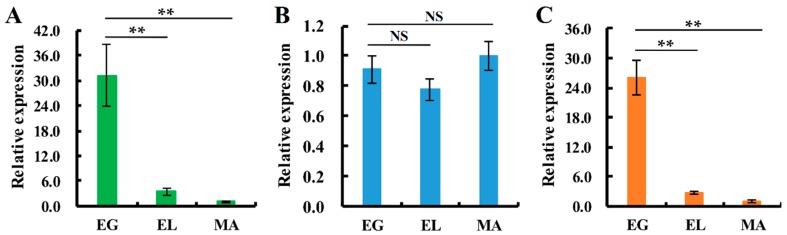
Expression patterns of *sod* genes in lamellae during different developmental stages of *V. volvacea* H1521. (**A**) the differential expression pattern of *Cu-Znsod1* gene; (**B**): the differential expression pattern of *Mnsod1* gene; and (**C**) the differential expression pattern of *Mnsod2* gene. EG = egg stage; EL = elongation stage; MA = maturation stage. The gene expression levels are presented relative to the lamellae of MA stage. The means of four independent experiments are shown. The error bars show the standard error of the mean. Statistical analysis was performed using LSD test, ** *p* < 0.01, NS means no signiﬁcant differences.

All three SOD-encoding genes showed significantly different levels of expression in different growth stages of the fruiting body of *V. volvacea*. The statistical analysis indicated that the expression patterns of *Vv_Cu-Znsod1* and *Vv_Mnsod2* show similar expression patterns in different tissues of *V. volvacea*. The correlation coefficient, 0.949, indicates a very good correlation [25]. Both genes show the highest level of expression in the pileus of the egg stage and are thought to be correlated with the morphogenesis of the pileus at this stage. However, the *Vv_Mnsod1* showed to be less tissue-specific since the highest expression tissue (button stage fruiting body) was only 5.6-fold than the lowest expression tissue (the stipe of elongation stage). Although, the expression level in the pileus was higher than in the stipe, the expression pattern of *Vv_Mnsod1* was different from the other two *sod* genes (correlation coefficients of 0.385 and 0.421) (Figure 5). Furthermore, the expression of *Vv_Cu-Znsod1* and *Vv_Mnsod2* in the lamellae showed good correlation with each other (correlation coefficient of 1), and the level of expression in the egg stage was much higher than in the elongation stage or the maturation stage. However, the expression of *Vv_Mnsod1* in the lamellae of egg stage showed no significant differences with elongation stage and maturation stage. (Figure 6).

## 3. Discussion

Alternative splicing was first described by Walter Gilbert in 1978 and was believed to be one of the important reasons for the functional diversity of proteins [26,27]. Lin *et al.* [28] cloned six different *sod* splice variants from longan somatic embryos and thought they could lead to the diversity of SOD sequences and functions. By mapping the reads obtained from the *V. volvacea* mixed sample transcriptome sequencing with the gene sequences, we found the *Vv*-*sod* genes also possess two alternative splicing modes of intron retention and 3’-terminal alternative splicing in the growth and development process of *V. volvacea*. By randomly combining with these alternative splicing modes, five potential Cu-Znsod1, four potential Mnsod1 and ten potential Mnsod2 amino acid sequences with different lengths and sequence motifs could be obtained (supplementary Figure S3). Chen *et al.* (2014) [29] isolated two different transcripts of 6-phosphogluconate dehydrogenase encoding gene in *V. volvacea* caused by sixth intron retention alternative splicing by using RT-PCR method, and the results suggest that both transcripts could be detected in the same sample, but with different expression levels. We guessed that *V. volvacea* also can indirectly or directly cause the increase, decrease or even the disappearance of SOD enzyme activity, by changing the content of the different transcripts *in vivo*. In addition, the results of Li *et al.* [19] showed that the *V. volvacea* can generate a maximum of eight SOD isozyme bands during low-temperature stress. *V. volvacea* only possesses three SOD encoding genes, and the other isozyme bands could be the alternative splicing products of the *sod* genes.

Tao *et al.* (2015) [30] found that the fifth intron of glucose-6-phosphate dehydrogenase encoding gene in *V. volvacea* contained a termination codon which could be retained during transcription process and made the conserved domain incomplete. In this study, intron I4 of the *Vv_Cu-Znsod1* contains one initiation codon. Only when I4 is retained can the sequence be translated into a functional Cu-ZnSOD amino acid sequence. This means that whether I4 is retained or not directly determines whether cells encode the Cu-ZnSOD or not. Such a special alternative splicing mode is rarely observed in the edible mushrooms or even in other living organisms. This finding provides important evidence that alternative splicing regulates functional Vv_Cu-ZnSOD1 during fruiting body development of *V. volvacea*.

The Cu-ZnSODs can be classified into two groups, intracellular Cu-ZnSOD (icCu-ZnSOD) and extracellular Cu-ZnSOD (ecCu-ZnSOD), according to the locations of the proteins [31]. Only ecCu-ZnSOD was found in decapod crustaceans; only icCu-ZnSOD was found in bacteria, fungi (although some Saccharomycetes, e.g., *Histoplasma capsulatum* contain both icCu-ZnSOD and ecCu-ZnSOD), plants, platyhelminths, molluscs and fish; and both icCu-ZnSOD and ecCu-ZnSOD were found in cnidarians, nematodes, insects, and mammals [4,32,33,34,35,36]. This study identified an ecCu-ZnSOD encoding gene in the basidiomycetes (*V. volvacea*) for the first time, whereas no icCu-ZnSOD encoding gene was found, which means that the antioxidant system of *V. volvacea* is different from that of other fungi.

To avoid missing some of the genes that encode SOD because of splicing errors, we downloaded the genome data [37] of *V. volvacea* V23 from the Joint Genome Institute (JGI) fungi portal (http://genome.jgi.doe.gov/programs/fungi/index.jsf) and identified the SOD-encoding genes by the local BLASTP method. The findings indicated that only one ecCu-ZnSOD encoding gene was found in V23, and no icCu-ZnSOD encoding gene existed; in addition to MnSOD1 and MnSOD2, a suspected MnSOD3 (sequence No. 121275) was found in V23 but with only a partial SOD domain. After further analysis, we found the sequence GME5781 in PYd21 genome, which was highly similar to the suspected MnSOD3 sequence. Using Sanger method to verify the DNA sequence, then using the transcriptome reads to obtain the accurate transcripts. The amino acid sequence of the protein were predicted using the ORF finder and then submitted to the NCBI for structural domain prediction, as shown in Figure S4. This sequence contains a PIdB domain but no conserved SOD motif, and the suspected MnSOD3 was annotated because of the wrong predicted amino acid.

Many studies of the important role of the expression levels of the *sod* genes during the infection of a host by pathogenic fungi have been carried out [24,38,39,40,41]. However, there are only a few reports of the function of SOD proteins during fungus growth and development and response to abiotic stress [42,43]. This study used the RT-qPCR method to detect the expression of the three *sod* genes during the cold stress response and fruiting body growth and development. The results showed that all three *sod* genes participate in the fruiting body development and cold stress response in *V. volvacea*.

Interestingly, the results of the expression levels in different fruiting-body samples showed that the expression levels of the *Cu-Znsod1* and the *Mnsod2* in the pilei of the egg stage were significantly higher than in other samples; although not the highest, the expression level of *Mnsod1* in the egg stage of the pileus was significantly higher than in the stipe, and it gradually decreased during maturation. The pileus is the primary place for sexual propagation of *V. volvacea*. From the button stage, the lamellae start differentiating, and a series of sexual differentiation steps, including basidium formation, cell nucleus integration, meiosis and production of basidiospores occur on the lamellae. Li *et al.* [44] studied about the behaviour of cell nuclei during meiosis and basidiospore formation indicated that *V. volvacea* needs approximately 30 h from the differentiation of the lamellae to full maturity of the fruiting-body. During this process, approximately 80% of the basidia proceeded with karyogamy. By 3 h of the egg stage, 37.5% of the basidia had proceeded to karyogamy. However, only approximately 30% and 0.1% of the basidia had proceeded with karyogamy by 9 h of the elongation stage and 7 h of the maturation stage, respectively. The Pearson correlation coefficient method suggested that the efficiency of karyogamy was correlated with both Cu-Znsod1 and Mnsod2 gene expression (correlation coefficients of 0.983 and 0.981, respectively). Therefore, we speculated that the high expression levels of the *sod* genes in the pilei in the egg stage were associated with the regulation of karyogamy of the basidia.

The low temperature autolysis of the *V. volvacea* is always one of the major factors that impede the industrial development and product distribution of *V. volvacea*. Sun *et al.* [45] used mRNA differential display PCR to identify four cold inducible genes. Bao *et al.* [37] used the transcriptome sequencing method to detect the differential expression of these genes during low temperature induction at different times and obtained 25 genes that were up-regulated and 55 that were down-regulated. By analyzing the antioxidase isozyme electrophoreogram of *V. volvacea* under cold stress at different times, Wang *et al.* [46] found that the SOD of *V. volvacea* was involved in the cold response. The expression levels of the isozymes of SOD showed a pattern of increase followed by a decrease. In addition, the crucial free radical scavenger Cu-ZnSOD could defend against oxidative stress and protect the membrane from ROS damage [47]. However, in our study, we found that *V. volvacea* lacks icCu-ZnSOD, and the ecCu-ZnSOD can be encoded only when the first intron retained, which suggested a possible reason for the autolysis of *V. volvacea* under cold conditions. In the future, transforming the exogenous *icCu-Znsod* gene into *V. volvacea* may be conducive to improving the cold resistance.

## 4. Materials and Methods

### 4.1. Strains

The fungal strain H1521 (collection number ACCC52633) is a heterokaryon able to fruit normally and was formed by crossing PYd15 (ACCC52631) with PYd21 (ACCC52632), two homokaryon strains with opposite mating types. All three strains are now preserved in the Agricultural Culture Collection of China (ACCC) with preservation No. ACCC52633.

### 4.2. Genome and Transcriptome Sequencing Data

*De novo* sequencing of the whole genome of the single spore strain PYd21 as well as the transcriptome sequencing of balanced mixed mRNA of eight samples, *i.e.*, the mycelia of PYd15, PYd21 and H1521 and the primordium, button-stage stipe, egg-stage stipe, elongated-stage stipe, and maturation-stage stipe after fruiting of H1521, was performed on the Solexa/Illumina platform at the Shenzhen Huada Gene Research Institute. The draft genome of PYd21 was assembled using a SOAPdenovo assembler [48] and is available under accession no. PRJNA171553 at NCBI.

### 4.3. Identification of the SOD Proteins of V. volvacea

The reviewed amino acid sequences of Fe mitochondrial superoxide dismutase, Mn mitochondrial superoxide dismutase, Ni mitochondrial superoxide dismutase and Cu-Zn superoxide dismutase were downloaded from the UniProt database (http://www.uniprot.org/) and locally compared with the 11,534 amino acid sequences of *V. volvacea* [49] using BLASTP after a standardized library was constructed. Amino acid sequences with an *e*-value ≤ 1 × e^−2^ were extracted using Perl scripts and submitted to NCBI for domain prediction to eliminate the sequences without any SOD superfamily conserved domains.

### 4.4. Acquisition, Accuracy Verification and Structural Analysis of Full-Length Gene Sequences

Based on the method of Yan *et al.* [50], the acquisition, accuracy verification and structural analysis were performed by using the predicted SOD encoding DNA sequences of *V. volvacea* together with the 2000 bp upstream and downstream sequences as references to map the reads in 500 bp read pools of transcriptome sequencing using the ZOOM software [51]. Using the paired-end method to determine the transcription start site and termination site of the genes, the fragment between the transcription start site and termination site was considered to be the full-length gene sequence.

The full-length gene sequence and its upstream and downstream 500 bp was used as the reference sequence, and the ZOOM software was applied to map the reads in the 500 bp read pools obtained by genome sequencing with the paired end method to verify the accuracy of the sequence. It proved to be accurate if all the base pairs of a gene were covered by reads. If they were not, it indicated that the sequence acquired some errors during splicing or sequencing. All the gene sequences were sequenced again by the Sanger method to guarantee the accuracy of the sequences.

The verified sequence was used as the reference for remapping the reads obtained by the transcriptome sequencing by means of the paired end method and ZOOM software and for determining introns, exons, and alternative splicing sites according to the GT-AG rule. An open reading frame (ORF), a 5’ untranslated region (5’UTR) and a 3’ untranslated region (3’UTR) of the gene can be predicted from the website, ORF Finder.

The parameters of the ZOOM software were set as follow: the distance of the adjacent paired reads was 1–2000 bp, and the data format was the Illumina type. The tolerance for mismatched base pair(s) between the reference sequence and the reads was 40 bp and 0 bp for mapping the transcriptome and genome reads, respectively. The other parameters were set to the defaults. The detailed method of gene sequence verification and structural analysis can be observed in the supplementary method S1.

### 4.5. Bioinformatics Analysis

Multiple sequence alignment was performed using the MUSCLE method, and the neighbor-joining (NJ) phylogenetic tree was constructed using MEGA5.1 software [52]. The conserved domains of the amino acid sequences were coloured using GeneDoc software [53]. Subcellular localization of the proteins was performed using TargetP 1.1 (http://www.cbs.dtu.dk/services/TargetP) and MITOPROT (http://ihg.gsf.de/ihg/mitoprot.html), and signal peptides were predicted using SignalP 4.1 (http://www.cbs.dtu.dk/services/SignalP/) [54,55].

### 4.6. Cold Stress Treatment

The buffer solution was prepared according to the biological characteristics of the organism. Our preliminary test suggested that the mycelia suffered the least abiotic stress when they were at 34 °C and the pH value was 8. Therefore, we chose 0.02 mol/L PBS (phosphate buffered saline) (pH 8) as the incubation buffer used for unstressed mycelia.

For cold stress, the mycelia of strain H1521 were inoculated on glass papers, which were placed directly on the surface of solid PDA plates (Φ = 6 cm), and cultured at 34 °C in the dark for three days. When the plates were fully covered in colonies, the cold stress test was performed. To prevent the interference of other stresses, about 10 mL of sterile PBS buffer (0.02 mol/L, pH 8, 34 °C) was first added to each plate to completely submerge the colonies in the buffer and the plates were incubated at 34 °C, followed by exchanging the warm PBS buffer with cold PBS buffer (4 °C) and incubating at 4 °C. Hyphae incubated in sterile PBS buffer (0.02 mol/L, pH 8) at 34 °C for 1.5 h was used as the control.

After treatment, mycelia were quickly scraped from the plates, blot-dried and stored at −80 °C for RNA extraction after removing the incubation solution and the inoculation agar blocks. All treatments were repeated in at least four independent experiments.

### 4.7. Mycelia and Fruiting-Body Growth and Sample Collection

The strain PYd21 was cultured in liquid PDA medium at 34 °C. After nine days, the mycelia were collected and stored at −80 °C for DNA extraction. The strain H1521 was used for producing fruiting bodies according to the Chen’s method [56]. A total of 11 samples were collected, including primordia stage fruiting bodies (PR), button stage fruiting bodies (BU), the stipe, pileus and lamellae of the egg stage (EG-stipe, EG-pileus, EG-lamellae), the stipe, pileus, and lamellae of the elongation stage (EL-stipe, EL-pileus, EL-lamellae), and the stipe, pileus, and lamellae of the maturation stage (MA-stipe, MA-pileus, MA-lamellae). In terms of the standard of Tao *et al.* [57], at least three independent replicates were collected for each sample and immediately frozen in liquid nitrogen for RNA extraction. 

### 4.8. DNA Extraction, PCR Amplification, and Sequence Determination

Using the cetyltrimethyl ammonium bromide (CTAB) method [58], genomic DNA was extracted from PYd21 mycelia; the PCR amplification was conducted on a BIO-REDS1000™ Thermal Cycler PCR amplifier (Bio-Rad Laboratories, Inc., Hercules, CA, USA), and the primer sequences and the annealing temperatures are listed in Table 1.

**Table 1:** Gene-specific primers used to amplify the full-length DNA sequences.

Gene	Primer Sequence（5’-3’）	Annealing Temperature for PCR
*Vv_Cu-Znsod1*	GGTTGCCGTCCTGTCCTTCTT	58.0 °C
	CGCCTGTAGTATTCTTGCCCTCAT	
*Vv_Mnsod1*	GACCAACGACCTTGATCCATC	54.6 °C
	GGACAACCAACGCCTGATG	
*Vv_Mnsod2*	GGTGAGAGTTGGAAGCGGTA	54.9 °C
	GGCACTGGACAGAGCGTAT	
GME5781	TTCCTTTACGCTGCCAACT	55.4 °C
	GTACGGCTGTGAATATGTCGA	

### 4.9. RNA Extraction and Reverse Transcription 

The total RNA of the samples was extracted using the E.Z.N.A.™ Plant RNA kit (from Omega Bio-Tek, Norcross, GA, USA) according to the common sample extraction method. The first strand cDNA was synthesized using the TransScript All-in-one First-Strand cDNA Synthesis SuperMix for qPCR (One Step gDNA Removal) kit (from TransGen Biotech, Beijing, China) and the TransScript One-Step gDNA Removal and cDNA Synthesis SuperMix kit (from TransGen Biotech) according to the manufacturer’s instructions.

### 4.10. RT-qPCR Assay of Gene Expression

The RT-qPCR assay was performed with a quantitative reagent kit, TransStart Top Green qPCR SuperMix (from TransGen Biotech, Beijing, China) and the CFX real-time RT-qPCR amplifier (Bio-Rad Laboratories, Inc.). The PCR reaction systems and procedures were conducted according to the manufacturer's instructions, and qPCR was performed using the two-step method to improve specific amplification. The annealing temperature was 60 °C and the cycle number was 40; the relative expression of the gene was calculated using the 2^−ΔΔ*C*t^ method [59]. Glyceraldehyde-3-dehydrogenase encoding gene (*gapdh*) was used as the reference gene. The qPCR primers were designed using Primer premier 6.0 and synthesized by Sangon Biotech Co., Ltd. (Shanghai, China). See Table 2 for the primer sequences.

**Table 2:** Primers used in real-time quantitative PCR.

Gene	Forward Sequence (5’-3’)	Reverse Sequence (5’-3’)
*Vv_Cu-Znsod1*	ACATTCGTCGCTGCTCTCCT	GTGAAGTTGACTGTGCCTGTGA
*Vv_Mnsod1*	CGTTACCACCGCCAACCAAGA	TTAGGAGAGACCCAACCCTTAAAGC
*Vv_Mnsod2*	TGCTCGCCATCGCCAGAACT	GGTGGTGCTTGGTGTGGTGAAG
*gapdh*	CATCTTCCACTGGTGCGGCTAAG	GGCTTCTCAAGGCGAACGACAA

### 4.11. Statistical Analysis

The significance of gene expression was analyzed using SPSS Statistics v20 software. The correlations of different gene expression patterns was analyzed using the Pearson correlation coefficient method, and a histogram was drawn using Excel 2013.

## Supplementary Materials

Supplementary materials can be found at http://www.mdpi.com/1422-0067/17/1/34/s1.

## References

[1] Sun W., Shi Z., Zhang G. (2013). Research progress of superoxide dismutase. J. Mod. Agric..

[2] Miller A.F. (2012). Superoxide dismutases: Ancient enzymes and new insights. FEBS Lett..

[3] Bannister J.V., Bannister W.H., Rotilio G. (1987). Aspects of the structure, function, and applications of superoxide dismutase. Crit. Rev. Biochem. Mol. Biol..

[4] Bafana A., Dutt S., Kumar A., Kumar S., Ahuja P.S. (2011). The basic and applied aspects of superoxide dismutase. J. Mol. Catal. B Enzym..

[5] Dong L., He Y., Wang Y., Dong Z. (2013). Research progress on application of superoxide dismutase (SOD). J. Agric. Sci. Technol..

[6] Oberley L.W., Oberley T.D., Buettner G.R. (1980). Cell differentation, aging and cancer: The possible roles of superoxide and superoxide dismutases. Med. Hypotheses.

[7] Clair D.K.S., Oberley T.D., Muse K.E., Clair W.H.S. (1994). Expression of manganese superoxide dismutase promotes cellular differentiation. Free Radic. Biol. Med..

[8] Zelko I.N., Mariani T.J., Folz R.J. (2002). Superoxide dismutase multigene family: A comparison of the CuZn-SOD (SOD1), Mn-SOD (SOD2), and EC-SOD (SOD3) gene structures, evolution, and expression. Free Radic. Biol. Med..

[9] Landis G.N., Tower J. (2005). Superoxide dismutase evolution and life span regulation. Mech. Ageing Dev..

[10] Kim B.M., Rhee J.S., Park G.S., Lee J., Lee Y.M., Lee J.S. (2011). Cu/Zn-and Mn-superoxide dismutase (SOD) from the copepod Tigriopus japonicus: Molecular cloning and expression in response to environmental pollutants. Chemosphere.

[11] Wang Q., Yuan Z., Wu H., Liu F., Zhao J. (2013). Molecular characterization of a manganese superoxide dismutase and copper/zinc superoxide dismutase from the mussel Mytilus galloprovincialis. Fish Shellfish Immunol..

[12] Umasuthan N., Bathige S.D.N.K., Thulasitha W.S., Qiang W., Lim B.S., Lee J. (2014). Characterization of rock bream (Oplegnathus fasciatus) cytosolic Cu/Zn superoxide dismutase in terms of molecular structure, genomic arrangement, stress-induced mRNA expression and antioxidant function. Comp. Biochem. Physiol. Part B Biochem. Mol. Biol..

[13] Jia H., Sun R., Shi W., Yan Y., Li H., Guo X., Xu H. (2014). Characterization of a mitochondrial manganese superoxide dismutase gene from Apis cerana cerana and its role in oxidative stress. J. Insect Physiol..

[14] Chang S.T., Buswell J.A. (1996). Mushroom nutriceuticals. World J. Microbiol. Biotechnol..

[15] Reyes R.G. (2000). Indoor cultivation of paddy straw mushroom, *Volvariella volvacea*, in crates. Mycology.

[16] Zhao F., Lin J., You L., Guo L. (2010). Exploration on industrialized cultivation pattern of *Volvariella volvacea*. Edible Fungi China.

[17] Chen M., Yu Z., Fan P., Ling X., Wang N., Tan Q., Lu Z., Pan Y. (2000). Study on the Genetic Variation in tissue isolates of *Volvariella volvacea*. Acta Edulis Fungi.

[18] Liu Z., Zhang K., Lin J.F., Guo L.Q. (2011). Breeding cold tolerance strain by chemical mutagenesis in *Volvariella volvacea*. Sci. Hortic..

[19] Li J., Zhu Z. (1987). A study on the superoxide dismutase of the fruitbody of straw mushroom, *Volvariella volvacea*. J. Fujian Agric. Coll..

[20] Li J., Zhu J., Guo S. (1990). The Superoxide Dismutase of the mycelia of straw mushroom, *Volvariella volvacea*. J. Fujian Agric. Coll..

[21] Belinky P.A., Goldberg D., Krinfeld B., Burger M., Rothschild N., Cogan U., Dosoretz C.G. (2002). Manganese-containing superoxide dismutase from the white-rot fungus *Phanerochaete chrysosporium*: Its function, expression and gene structure. Enzym. Microb. Technol..

[22] Yin C., Zhao W., Zheng L., Chen L., Tan Q., Shang X., Ma A. (2014). High-level expression of a manganese superoxide dismutase (PoMn-SOD) from *Pleurotus ostreatus* in *Pichia pastoris*. Appl. Biochem. Biotechnol..

[23] Lamarre C., LeMay J.D., Deslauriers N., Bourbonnais Y. (2001). Candida albicans expresses an unusual cytoplasmic manganese-containing superoxide dismutase (*SOD3* gene product) upon the entry and during the stationary phase. J. Biol. Chem..

[24] Fréalle E., Noël C., Nolard N., Symoens F., Felipe M.S., Dei-Cas E., Camus D., Viscogliosi E., Delhaes L. (2006). Manganese superoxide dismutase based phylogeny of pathogenic fungi. Mol. Phylogenet. Evol..

[25] Eriksson M., Lindström B. (2005). Validity of Antonovsky’s sense of coherence scale: A systematic review. J. Epidemiol. Community Health.

[26] Graveley B.R. (2001). Alternative splicing: Increasing diversity in the proteomic world. Trends Genet..

[27] Roberts G.C., Smith C.W.J. (2002). Alternative splicing: Combinatorial output from the genome. Curr. Opin. Chem. Biol..

[28] Lin Y.L., Lai Z.X. (2013). Superoxide dismutase multigene family in longan somatic embryos: A comparison of *CuZn-SOD*, *Fe-SOD*, and *Mn-SOD* gene structure, splicing, phylogeny, and expression. Mol. Breed..

[29] Chen Z., Fu M., Li Y., Tao Y., Jiang Y., Xie B. (2014). Cloning and expression of 6-phosphogluconate dehydrogenase alternative splicing in *Volvariella volvacea*. Chin. J. Appl. Environ. Biol..

[30] Tao Y., Zhang L., Guo L., Chen R., Lian L., Xie B. (2015). Cloning and expression analysis of glucose-6-phosphate dehydrogenase gene and alternative splicing variant in *Volvariella volvacea*. Mycosystema.

[31] Fattman C.L., Schaefer L.M., Oury T.D. (2003). Extracellular superoxide dismutase in biology and medicine. Free Radic. Biol. Med..

[32] Lin Y.C., Vaseeharan B., Chen J.C. (2008). Identification of the extracellular copper–zinc superoxide dismutase (ecCuZnSOD) gene of the mud crab *Scylla serrata* and its expression following β-glucan and peptidoglycan injections. Mol. Immunol..

[33] Holbrook E.D., Edwards J.A., Youseff B.H., Rappleye C.A. (2011). Definition of the extracellular proteome of pathogenic-phase *Histoplasma capsulatum*. J. Proteome Res..

[34] Meng Q., Du J., Yao W., Xiu Y., Li Y., Gu W., Wang W. (2011). An extracellular copper/zinc superoxide dismutase (ecCuZnSOD) from Chinese mitten crab, *Eriocheir sinensis* and its relationship with *Spiroplasma eriocheiris*. Aquaculture.

[35] Youseff B.H., Holbrook E.D., Smolnycki K.A., Rappleye C.A. (2012). Extracellular superoxide dismutase protects *Histoplasma* yeast cells from host-derived oxidative stress. PLoS Pathog..

[36] Xiu Y., Wu T., Du J., Yao W., Li W., Ding Z., Ren Q., Gu W., Meng Q., Wang W. (2013). Molecular characterization and expression analysis of extracellular copper/zinc superoxide dismutase (ecCuZnSOD) from oriental river prawn, *Macrobrachium nipponense*. Aquaculture.

[37] Bao D., Gong M., Zheng H., Chen M., Zhang L., Wang H., Jiang J., Wu L., Zhu Y., Zhu G. (2013). Sequencing and comparative analysis of the straw mushroom (*Volvariella volvacea*) genome. PLoS ONE.

[38] Takao S., Smith E.H., Wang D., Chan C.K., Bulkley G.B., Klein A.S. (1996). Role of reactive oxygen metabolites in murine peritoneal macrophage phagocytosis and phagocytic killing. Am. J. Physiol. Cell Physiol..

[39] Karlsson M., Stenlid J., Olson Å. (2005). Identification of a superoxide dismutase gene from the conifer pathogen *Heterobasidion annosum*. Physiol. Mol. Plant Pathol..

[40] Lambou K., Lamarre C., Beau R., Dufour N., Latge J.P. (2010). Functional analysis of the superoxide dismutase family in *Aspergillus fumigatus*. Mol. Microbiol..

[41] Veluchamy S., Williams B., Kim K., Dickman M.B. (2012). The CuZn superoxide dismutase from *Sclerotinia sclerotiorum* is involved with oxidative stress tolerance, virulence, and oxalate production. Physiol. Mol. Plant Pathol..

[42] González-Guerrero M., Oger E., Benabdellah K., Azcón-Aguilar C., Lanfranco L., Ferrol N. (2010). Characterization of a CuZn superoxide dismutase gene in the arbuscular mycorrhizal fungus *Glomus intraradices*. Curr. Genet..

[43] Hwang C.S., Baek Y.U., Yim H.S., Kang S.O. (2003). Protective roles of mitochondrial manganese containing superoxide dismutase against various stresses in *Candida albicans*. Yeast.

[44] Li X.Y., Yan Y.Z., Shen R.D. (1991). Meiosis and behavior of nuclei during basidiospore formation in *Volvariella volvacea*. Acta Microbiol. Sin..

[45] Sun X., Feng A., Chen M., Pan Y.J. (2005). Cloning and sequence analysis of cold induced genes in Chinese straw mushroom, *Volvariella volvacea*. Mycosystema.

[46] Wang S., Zhang H., Cheng Q., He Q., He H. (2009). Effect of chilling stress on the expression of antioxidative enzymes in *Volvariella volvacea* mycelia. Chin. J. Trop. Crop..

[47] Wu D.F., Cederbaum A.I. (2003). Alcohol, oxidative stress, and free radical damage. Alcohol Res. Health.

[48] Li R., Zhu H., Ruan J., Qian W., Fang X., Shi Z., Li Y., Li S., Shan G., Kristiansen K. (2010). *De novo* assembly of human genomes with massively parallel short read sequencing. Genome Res..

[49] Meng L., Yan J., Xie B., Li Y., Chen B., Liu S., Li D., Yang Z., Zeng X., Deng Y. (2013). Genes encoding FAD-binding proteins in *Volvariella volvacea* exhibit differential expression in homokaryons and heterokaryons. Microbiol. Res..

[50] Yan J., Guo L., Zhao J., Xie B. (2014). Sequence characterization and differential expression of a glutathione *S*-transferase gene *vv-gto1* from *Volvariella volvacea*. Acta Microbiol. Sin..

[51] Zhang Z., Lin H., Ma B. (2010). ZOOM Lite: Next-generation sequencing data mapping and visualization software. Nucleic Acids Res..

[52] Tamura K., Peterson D., Peterson N., Stecher G., Nei M., Kumar S. (2011). MEGA5: Molecular evolutionary genetics analysis using maximum likelihood, evolutionary distance, and maximum parsimony methods. Mol. Biol. Evol..

[53] Nicholas K.B., Nicholas H.B., Deerfield D.W. (1997). GeneDoc: Analysis and visualization of genetic variation. Embnet. News.

[54] Claros M.G., Vincens P. (1996). Computational method to predict mitochondrially imported proteins and their targeting sequences. Eur. J. Biochem..

[55] Emanuelsson O., Nielsen H., Brunak S., von Heijne G. (2000). Predicting subcellular localization of proteins based on their N-terminal amino acid sequence. J. Mol. Biol..

[56] Chen S., Ge W., Buswell J.A. (2004). Molecular cloning of a new laccase from the edible straw mushroom *Volvariella volvacea*: Possible involvement in fruit body development. FEMS Microbiol. Lett..

[57] Tao Y., Xie B., Yang Z., Chen Z., Chen B., Deng Y., Jiang Y., van Peer A.F. (2013). Identification and expression analysis of a new glycoside hydrolase family 55 exo-β-1,3-glucanase-encoding gene in *Volvariella volvacea* suggests a role in fruiting body development. Gene.

[58] Möller E.M., Bahnweg G., Sandermann H., Geiger H.H. (1992). A simple and efficient protocol for isolation of high molecular weight DNA from filamentous fungi, fruit bodies, and infected plant tissues. Nucleic Acids Res..

[59] Livak K.J., Schmittgen T.D. (2001). Analysis of relative gene expression data using real-time quantitative PCR and the 2^−ΔΔ*C*t^ method. Methods.

