# Supplementary material for: The Sequence Characteristics and Expression Models Reveal Superoxide Dismutase Involved in Cold Response and Fruiting Body Development in Volvariella volvacea
Source: Int J Mol Sci. 2015 Dec 29;17(1):34. doi: 10.3390/ijms17010034 (PMC4730280; doi:10.3390/ijms17010034)
Supplement: Supplementary file 1 [file ijms-17-00034-s001.pdf]

Supplementary Materials: The Sequence Characteristics and Expression Models Reveal Superoxide Dismutase Involved in Cold Response and Fruiting Body Development in *Volvariella volvacea*

Jun-Jie Yan, Lei Zhang, Rui-Qing Wang, Bin Xie, Xiao Li, Ren-Liang Chen, Li-Xian Guo and Bao-Gui Xie

1. Method S1. Using Transcriptome Data to Analyze the Gene Structure

We used transcriptome data to confirm the gene structure of *vv_sod* genes, including the location of the origin and terminus of a gene, exons, introns, and possible alternative splicing sites. By using the DNA sequences of all predicted *V. volvacea* SOD encoding sequences, together with the 2000 bp upstream and downstream sequences as references (the real origin and terminus sites were usually within 1000 bp away from the predicted sites) to map the reads in 500 bp read pools of transcriptome sequencing using the ZOOM software [51]. We obtained 26,755,558 high-quality reads, including 2,408,000,220 nucleotides from transcriptome sequencing. The parameters of the software ZOOM Studio were as follows: the organism is diploid; the read file is FASTQ format (the FASTQ format will be regarded as the Illumina type); allow a maximum of 40 mismatched base pairs.

1.1. The Method to Confirm the Origin and Terminus Sites of Gene

In general, we can find the pair-end reads in one gene, *i.e.*, Gene A and B in the Figure M-1. However, no reads supported the spacer region between gene A and B. In addition, the pair-end reads mapped to gene A could not be found among the reads mapped to gene B. Therefore, we confirmed the origin and terminus sites through the spacer region of two genes. Here, we can confirm the origin and terminus sites of *vv_sod* genes according to this principle.

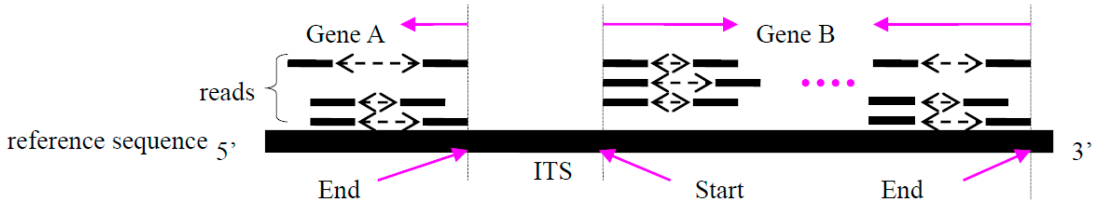

Figure M-1. The start and end sites of transcription. The horizontal black bars with double black dotted line arrow indicates a pair of mapping reads. The red dot means there are some other paired reads between the start and end sites of gene. ITS represents for internal transcribed spacer region between two genes.

1.2. The Method to Confirm the Intron Region

The intron region was determined according to the ZOOM software mapping result and the GT-AG rule. The method used to estimate the intron region was performed as Figure M-2.

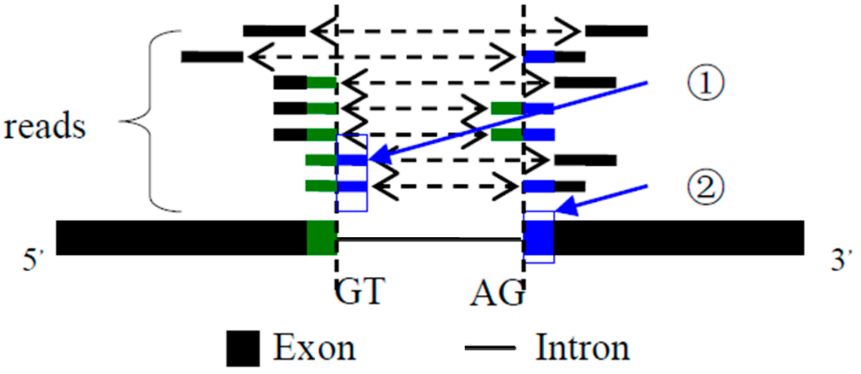

Figure M-2. The method to estimate intron region. The double arrow stand for a pair of reads. The same colors indicate a complete match with the reference sequence, such as the read sequence pointed out by the blue arrow 1 matches with the blue region of reference sequence pointed out by the arrow 2. The GT, AG were conservative Bases of intron region. The reads with both blue and green colors could matched with two neighboring exons which could be used to judge the potential intron region.

1.3. Analysis of Alternative Splicing

Alternative 3′ splice sites and intron retention sites were detected in this study. The judgment methods used for alternative 3′ splice sites and intron retention sites are provided in Figures M-3 and M-4.

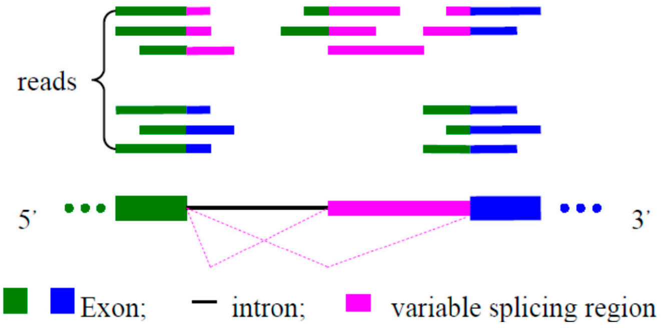

Figure M-3. The method used to estimate 3′ alternative splicing sites. The same colors indicate a complete match with the reference.

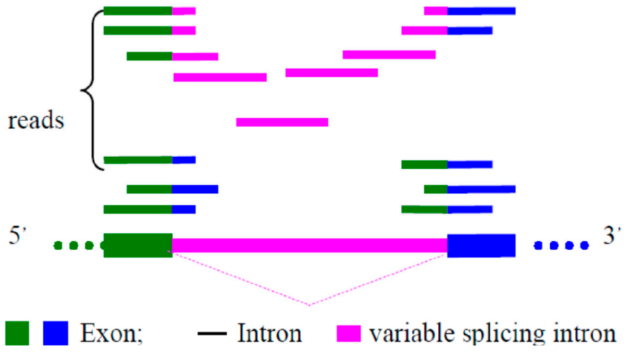

Figure M-4. The method used to estimate intron retention sites. The same colors indicate a complete match with the reference.

1.4. The ZOOM Software Results

The ZOOM software is designed to map millions of short reads, produced by next-generation sequencing technology, back to the reference genome, and carry out post-analysis in a user-friendly way. The detailed alignments of the mapped reads along the reference sequence is shown as Figure M-5:

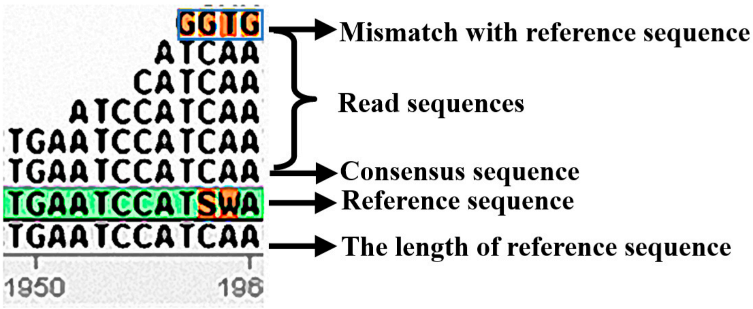

Figure M-5. The detailed alignments of the mapped reads along the reference sequence. Note: the sequence at the bottom of the window is the reference sequence. The sequence with green background over the reference sequence is the consensus sequence generated by the mapped reads along the reference sequence. The orange background of the nucleotides on the read or the consensus sequence highlights the difference from the nucleotide on the position of the reference sequence.

1.4.1. The *Vv_Cu-Znsod1* Gene Structure Judgment

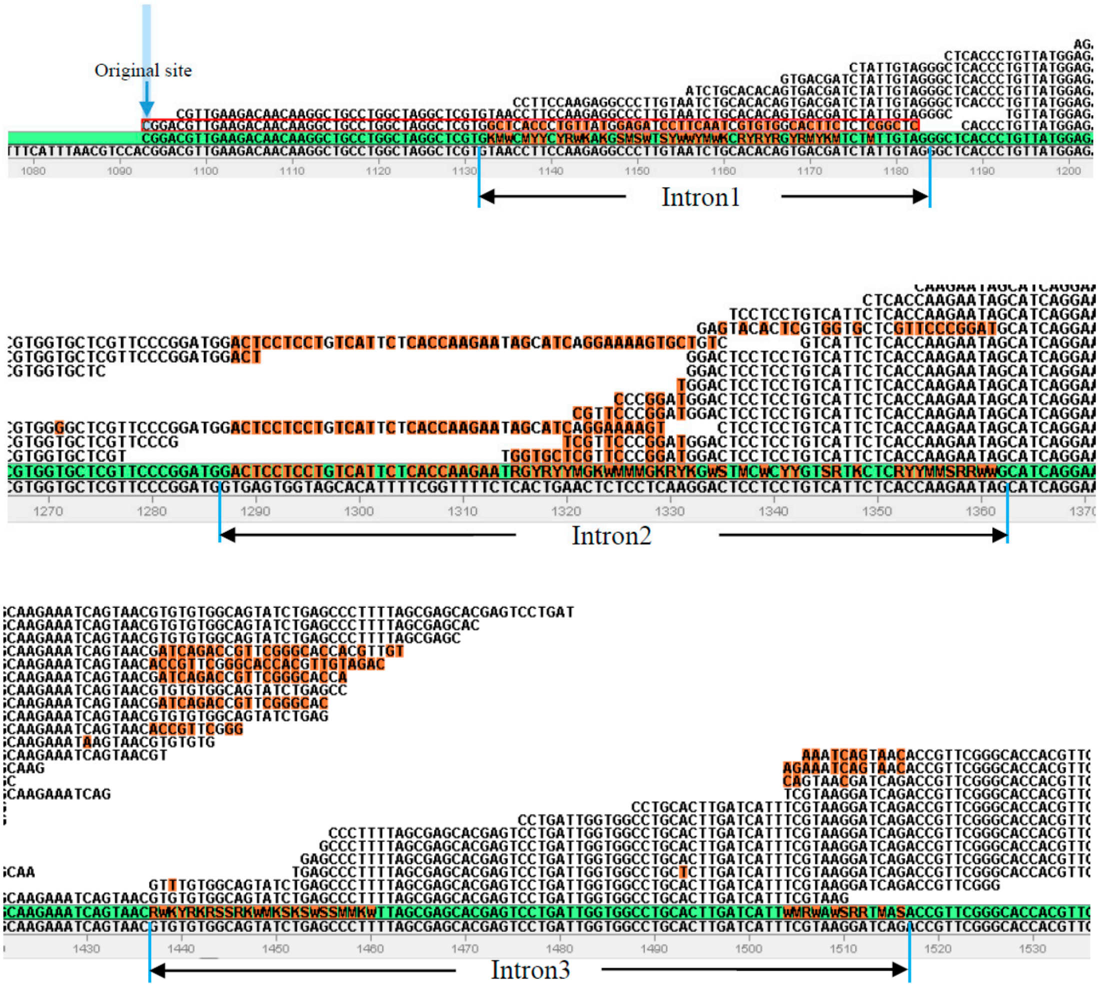

Figure M-6. *Cont.*

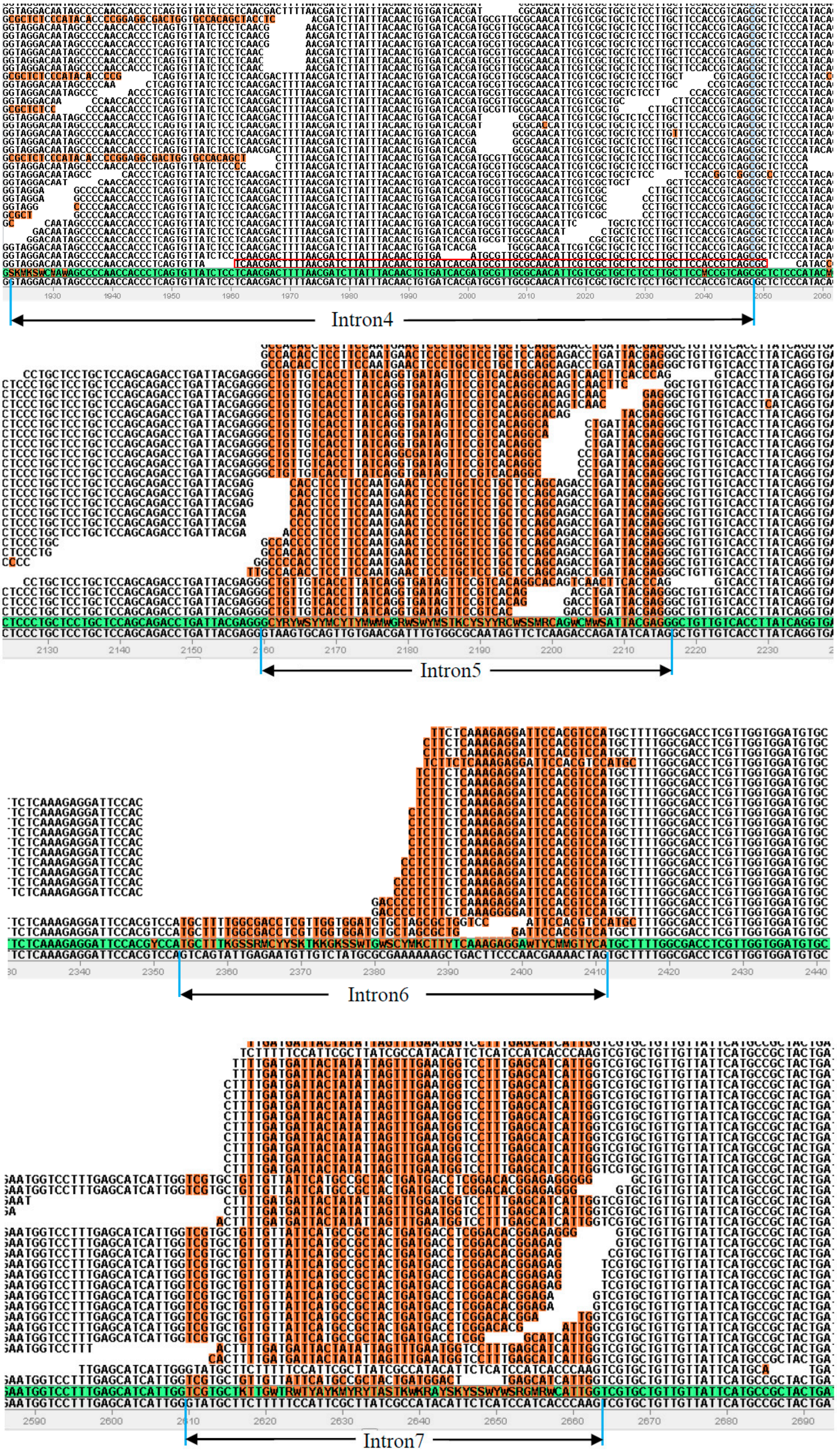

Figure M-6. *Cont.*

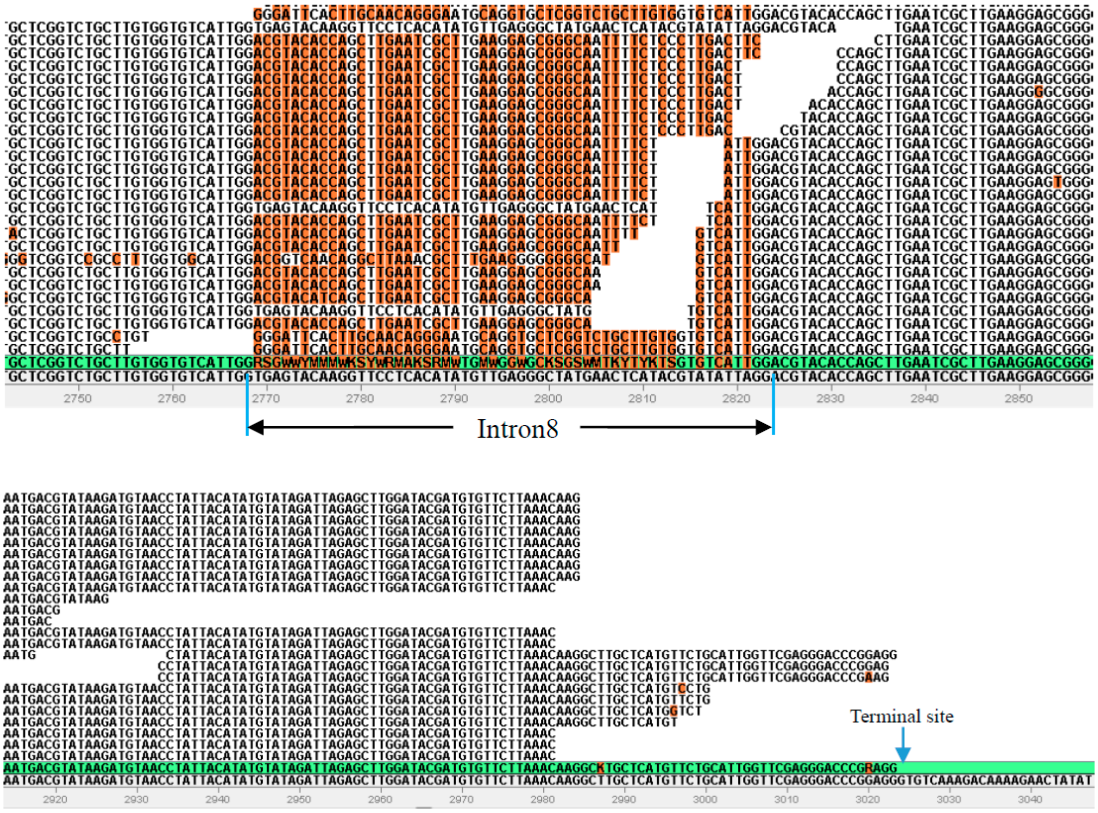

Figure M-6. The judgment of *Vv_Cu-Znsod1* gene structure. The sequence with green background over the reference sequence is the consensus sequence generated by the mapped reads along the reference sequence. The orange background of the nucleotides on the read or the consensus sequence highlights the difference from the nucleotide on the position of the reference sequence.

1.4.2. The *Vv_Mnsod1* Gene Structure Judgment

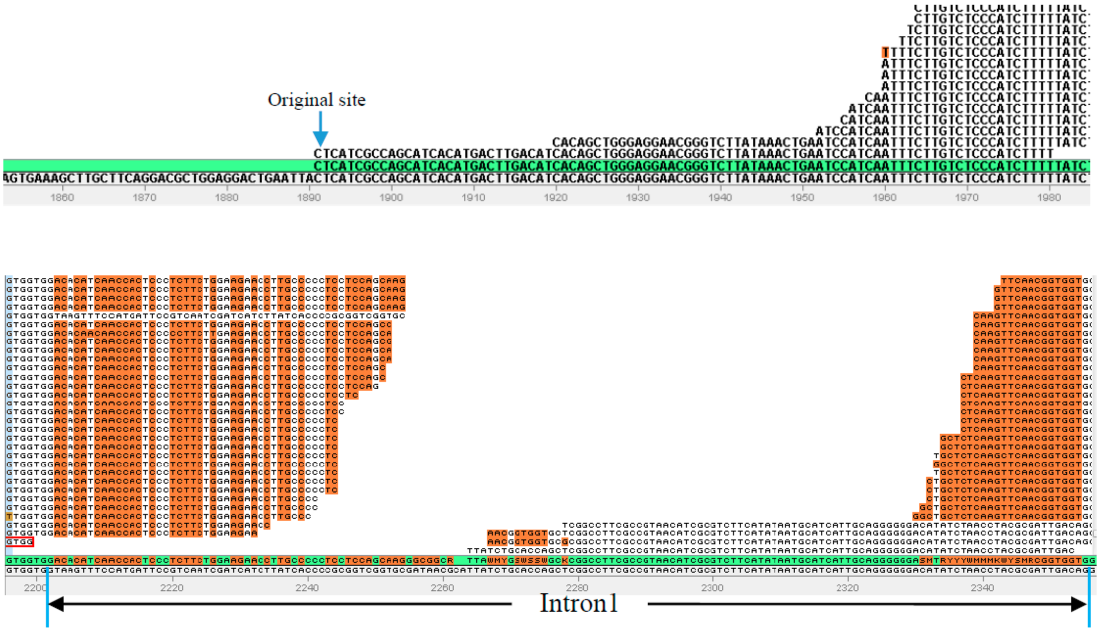

Figure M-7. *Cont.*

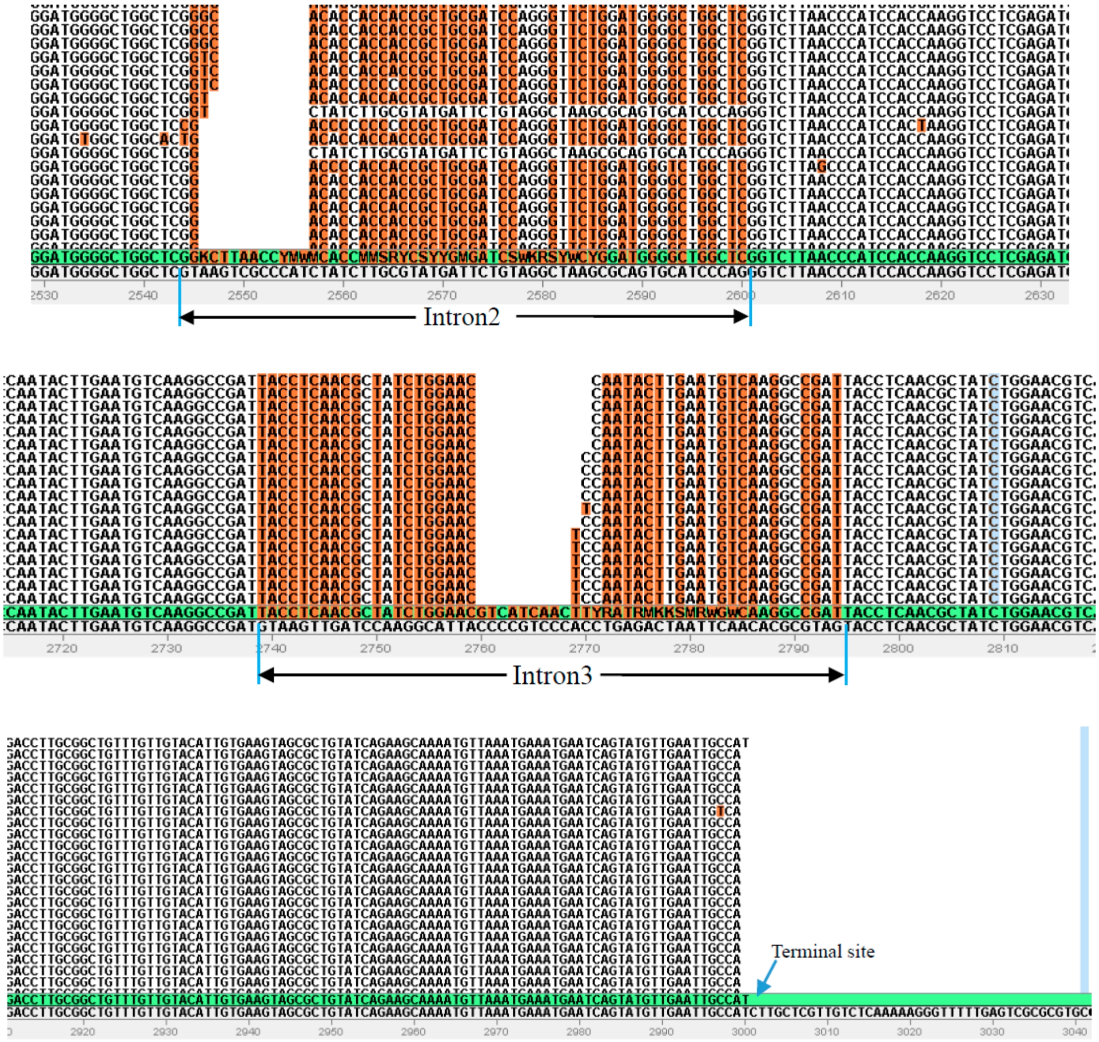

Figure M-7. The judgment of *Vv_Mnsod1* gene structure. The sequence with green background over the reference sequence is the consensus sequence generated by the mapped reads along the reference sequence. The orange background of the nucleotides on the read or the consensus sequence highlights the difference from the nucleotide on the position of the reference sequence.

1.4.3. The *Vv_Mnsod2* Gene Structure Judgment

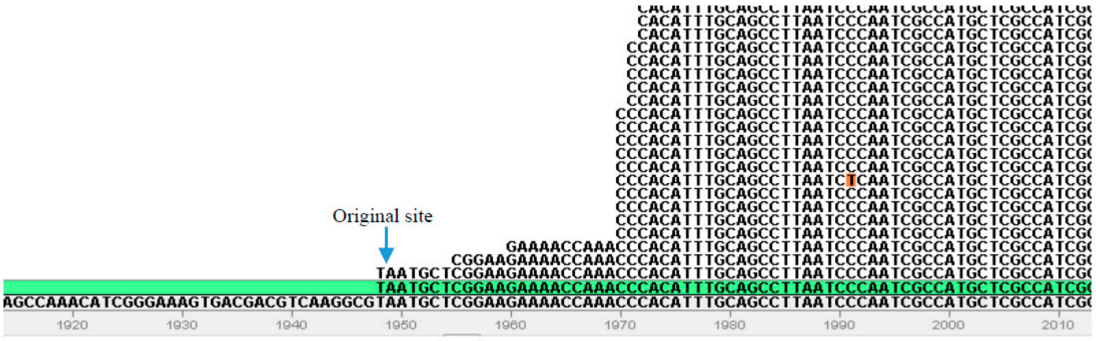

Figure M-8. *Cont.*

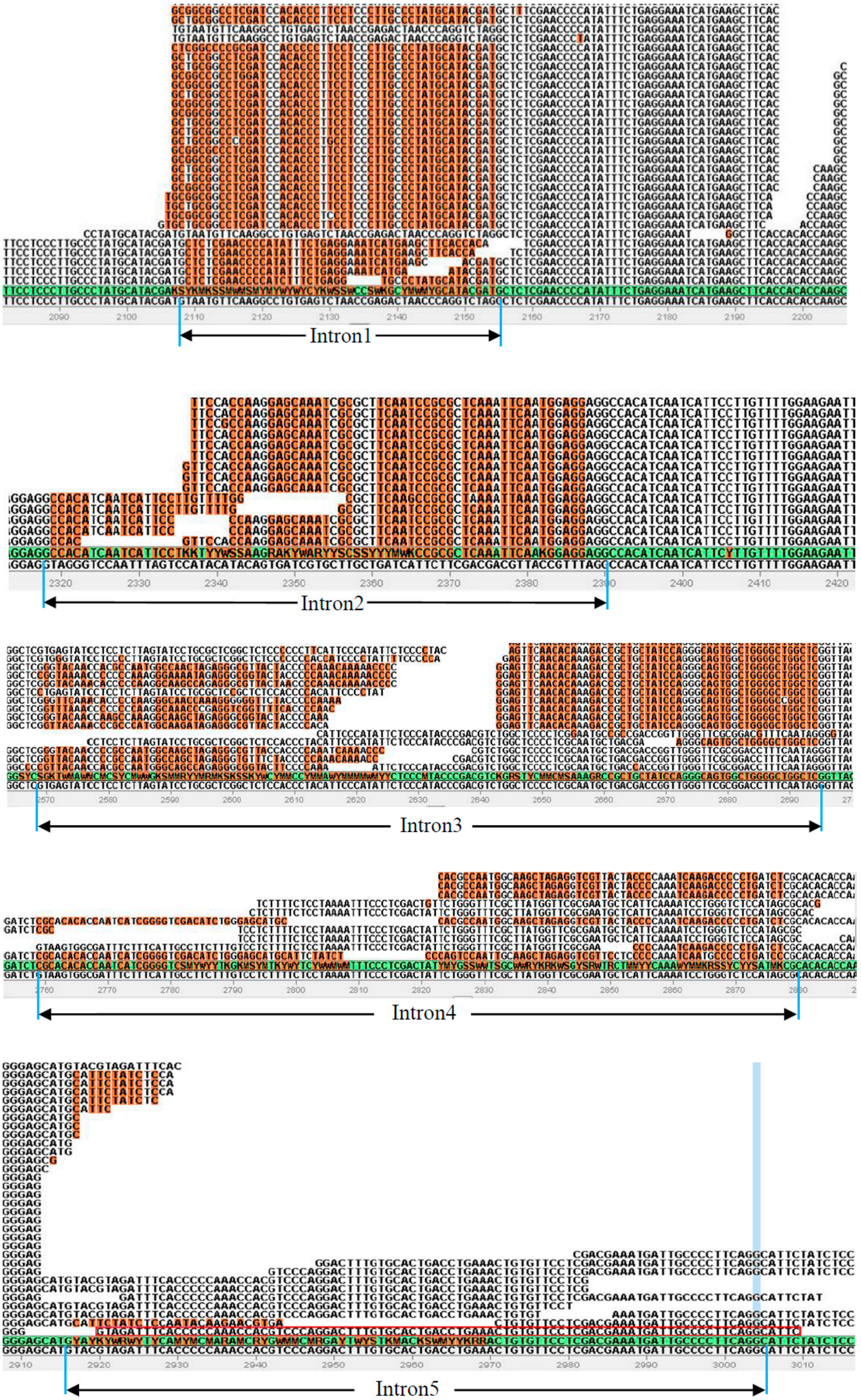

Figure M-8. *Cont.*

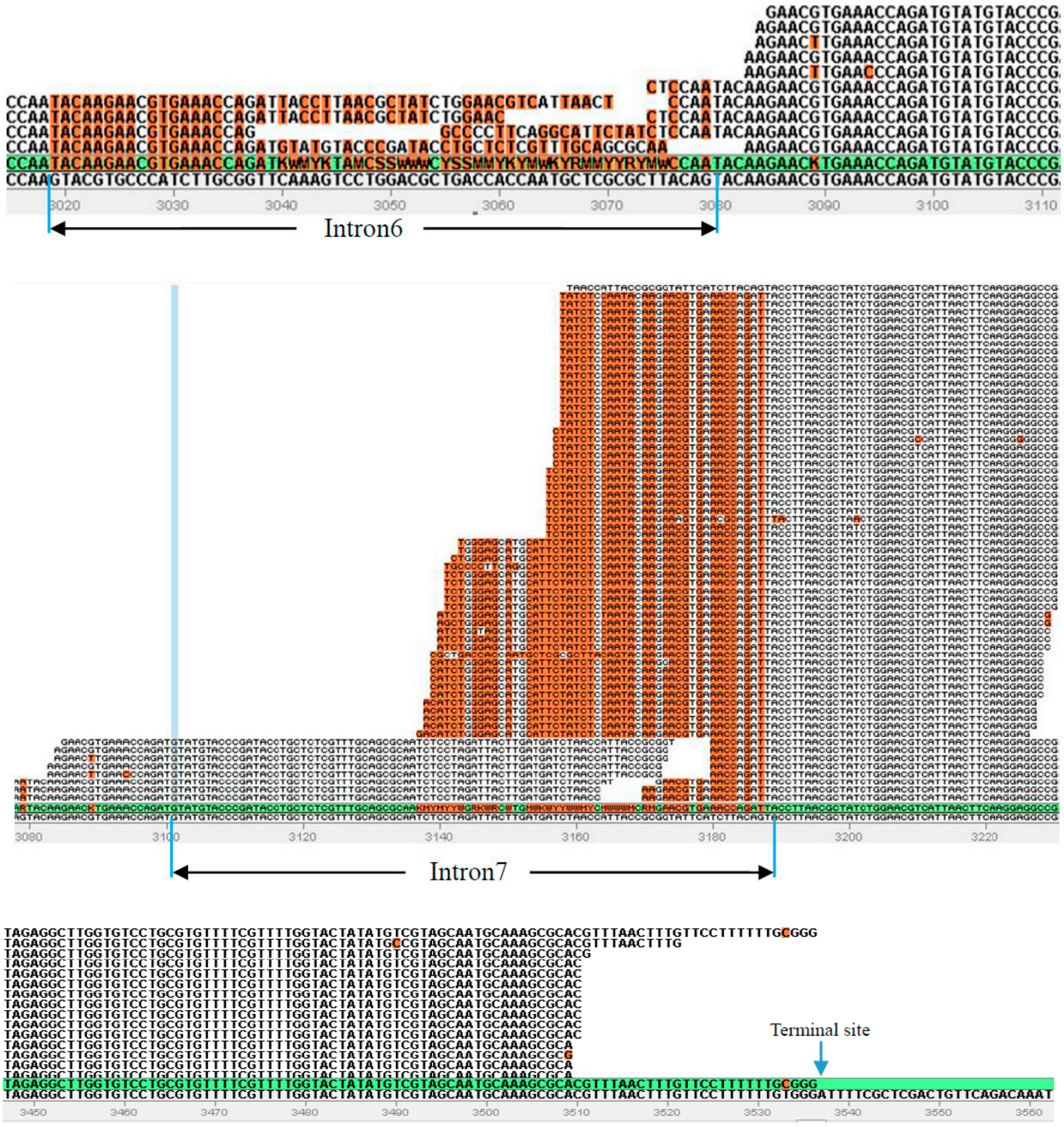

Figure M-8. The judgment of *Vv_Mnsod2* gene structure. The sequence with green background over the reference sequence is the consensus sequence generated by the mapped reads along the reference sequence. The orange background of the nucleotides on the read or the consensus sequence highlights the difference from the nucleotide on the position of the reference sequence.

2. Supplementary Sequences

The DNA sequence of the transcript region are shown as follows: the intron sequences are marked by red, the sequence with an underline indicates alternative splicing, the sequence with a double underline indicates that there are two possible alternative splice sites. The initiation codon and the termination codon are shown in boldface.

Sequence S1. *Vv_Cu-Znsod1* DNA sequence.
CGGACGTTGAAGACAACAAGGCTGCCTGGCTAGGCTCGTGTAACCTTCCAAGAGGCCCTTG
TAATCTGCACACAGTGACGATCTATTGTAGGGCTCACCCTGTTATGGAGATCCTTCAATCGTG
TGGCACTTCTCTCGGCTCTTGGCGTCCCCCAAGTCGTTCCGAGTACACTCGTGGTGCTCGTTC
CCGGATGGTGAGTGGTAGCACATTTTCGGTTTTCTCACTGAACTCTCCTCAAGGACTCCTCCT
GTCATTCTCACCAAGAATAGCATCAGGAAAAGTGCTGTCTGTGTTGAGTGCTCACGGTAAAT
ATAGGCAAAACGACTGGCAAGAAATCAGTAACGTGTGTGGCAGTATCTGAGCCCTTTTAGC

GAGCACGAGTCCTGATTGGTGGCCTGCACTTGATCATTTCGTAAGGATCAGACCGTTCGGGC
ACCACGTTGTAGACCCAATCATTTTATCTTGCAATGGGGCAACGCCACCCTGTTTTATTATCC
CGGGTAACGTCTTCGCGTATAGCCTAGAAGAAGCGAGGATGTAACTGCCTGCACATTTTTGC
ATTGTAAATGCCGCTTTGGGTGAGTGGCTCCCGTCTTTCCCGTCAGAAATAAAAGGTTTGTGT
CTCAAGTCTGAAGGGCGGTGAGGATCAATTGATGATGTCGCCGGACACGCCTCGAGCATGC
GTCACAATCATCCTTTTTAATTACGAAATGAATGGACTGGAACAAGGGCACAATGGAAGCC
ACGCCCGTTACATGCTCGGAGGCTGAAACGCTTCGTGACGCATGAATGGGCGCTCCATCATT
TAAAAACCTACATCTCAACCATGGTAGGACAATAGCCCCAACCACCCTCAGTGTTATCTCCT
CAACGACTTTTAACGATCTTATTTACAACTGTGATCACG**ATG**CGTTGCGCAACATTCGTCGCT
GCTCTCCTTGCTTCCACCGTCAGCGCTCTCCCATACACCCCGGAGGCGACTGGTGCCACAGC
TACCTCCACCATTGTTGCCACACCTCCTTCCAATGAACTCCCTGCTCCTGCTCCAGCAGACCT
GATTACGAGGGTAAGTGCAGTTGTGAACGATTTGTGGCGCAATAGTTCTCAAGACCAGATAT
CATAGGCTGTTGTCACCTTATCAGGTGATAGTTCCGTCACAGGCACAGTCAACTTCACCCAG
GAGAACTACGGAGGACCGGTGACAGTTAGTGGTCTAGTTCAAGGCCTTGACCCCTCTTCTCA
AAGAGGATTCCACGTCCAGTCAGTATTGAGAATGTTGTCTATGCGCGAAAAAAGCTGACTTC
CCAACGAAAACTAGTGCTTTTGGCGACCTCGTTGGTGGATGTGCTAGCGCTGGTCCTCACTT
CAATCCCTTCAACAGGACTCATGGTGACCGCTTGAGCAGTACCCGACACGTCGGTGATCTG
GGGAACGTTTTCAGCGACGAGAATGGTATCGCCACGTTCACTTTTGATGATTACTATATTAGT
TTGAATGGTCCTTTGAGCATCATTGGGTATGCTTCTTTTTCCATTCGCTTATCGCCATACATTCT
CATCCATCACCCAAGTCGTGCTGTTGTTATTCATGCCGCTACTGATGACCTCGGACACGGAG
AGGGGGATTCACTTGCAACAGGGAATGCAGGTGCTCGGTCTGCTTGTGGTGTCATTGGTGAG
TACAAGGTTCCTCACATATGTTGAGGGCTATGAACTCATACGTATATTAGGACGTACACCAGC
T**TGA**ATCGCTTGAAGGAGCGGGCAATTTTCTCCCTTGACTTCGGTGTTGATCGCGACGCTTGT
ATTTTATGACGGACAAATGACGTATAAGATGTAACCTATTACATATGTATAGATTAGAGCTTG
GATACGATGTGTTCTTAAACAAGGCTTGCTCATGTTCTGCATTGGTTCGAGGGACCCGGAGG

Sequence S2. *Vv_Mnsod1* DNA sequence

CTCATCGCCAGCATCACATGACTTGACATCACAGCTGGGAGGAACGGGTCTTATAAACTGAA
TCCATCAATTTCTTGTCTCCCATCTTTTTATCTTTGAACCAGCAACC**ATG**GCCCACACTCTCCC
TGATCTCCCATACGACTACAATGCTCTCGAGCCCTTCATCTCGGAGCAGATCATGACCCTGC
ACCACAAGAAACACCATCAGACTTACGTCAATGCCCTCAATGCAGCCGAGGAGGCATACGC
AAGGGCTTCCACCCCTAAGGAGCGCATCGCCCTCCAGGCTGCTCTCAAGTTCAACGGTGGT
GGTAAGTTTCCATGATTCCGTCAATCGATCATCTTATCACCCCGCGGTCGGTGCGATAACGCA
TTATCTGCACCAGCTCGGCCTTCGCCGTAACATCGCGTCTTCATATAATGCATCATTGCAGGG
GGGACATATCTAACCTACGCGATTGACAGGACACATCAACCACTCCCTCTTCTGGAAGAACC
TTGCCCCCTCCTCCAGCAAGGGCGGCAACGGTGGTGTTCTCAAGGATGGCCCCTTGAAGGA
CGCTATCATTCGCGCATTCGGAAGTGTTGAGGCCTTCAAGAAGGAGTTCAACACCACCACC
GCTGCGATCCAGGGTTCTGGATGGGGCTGGCTCGTAAGTCGCCCATCTATCTTGCGTATGATT
CTGTAGGCTAAGCGCAGTGCATCCCAGGGTCTTAACCCATCCACCAAGGTCCTCGAGATCGT
TACCACCGCCAACCAAGACCCTCTCCTCACCCACATCCCCATTATCGGTGTCGACATCTGGG
AGCACGCCTTCTACCTCCAATACTTGAATGTCAAGGCCGATGTAAGTTGATCCAAGGCATTA
CCCCGTCCCACCTGAGACTAATTCAACACGCGTAGTACCTCAACGCTATCTGGAACGTCATC
AACTTTGATGAGGCCGAGAAGCGCTTCACCGGCGAGTCTAAGCTT**TAA**GGGTTGGGTCTCTC
CTAAAAATCTGGTTTTTCGAGTTGGCGGACCTTGCGGCTGTTTGTTGTACATTGTGAAGTAGC
GCTGTATCAGAAGCAAAATGTTAAATGAAATGAATCAGTATGTTGAATTGCCAT

Sequence S3. *Vv_Mnsod2* DNA sequence

TAATGCTCGGAAGAAAACCAAACCCACATTTGCAGCCTTAATCCCAATCGCC**ATG**CTCGCCA
TCGCCAGAACTGCTCTACGCCCCGCCCTGTCTCGTCGTTTCGCAGCTCGTGCGGCGGCCTCG
ATCCACACCCTTCCTCCCTTGCCCTATGCATACGATGTAATGTTCAAGGCCTGTGAGTCTAAC
CGAGACTAACCCAGGTCTAGGCTCTCGAACCCCATATTTCTGAGGAAATCATGAAGCTTCAC

CACACCAAGCACCACCAGGCATACGTCAACGGCCTGAATGCAGCAGAGGAGGCCTACGCC
AAGACCAGTTCCACCAAGGAGCAAATCGCGCTTCAATCCGCGCTCAAATTCAATGGAGGAG
GTAGGGTCCAATTTAGTCCATACATACAGTGATCGTGCTTGCTGATCATTCTTCGACGACGTT
ACCGTTTAGGCCACATCAATCATTCCTTGTTTTGGAAGAATTTAGCTCCCGCCTCTGAAGACG
GCGGCAAATTGGCCGATGGGGCCCTCAAAAAGGCCATCGAACGAGACTTCGGCTCCGTGG
ACGCGTTCAAGAAGGAGTTCAACACAAAGACCGCTGCTATCCAGGGCAGTGGCTGGGGCT
GGCTCGTGAGTATCCTCCTCTTAGTATCCTGCGCTCGGCTCTCCACCCTACATTCCCATATTCT
CCCATACCCGACGTCTGGCTCCCCTCGCAATGCTGACGACCGGTTGGGTTCGCGGACCTTTC
AATAGGGTTACAACCACGCCAATGGCAAGCTAGAGGTCGTTACTACCCCAAATCAAGACCC
CCTGATCTGTAAGTGGCGATTTCTTTCATTGCCTTCTTTGTCCTCTTTTCTCCTAAAATTTCCCT
CGACTATTCTGGGTTTCGCTTATGGTTCGCGAATGCTCATTCAAAATCCTGGGTCTCCATAGC
GCACACACCAATCATCGGGGTCGACATCTGGGAGCATGTACGTAGATTTCACCCCCAAACC
ACGTCCCAGGACTTTGTGCACTGACCTGAAACTGTGTTCCTCGACGAAATGATTGCCCCTTC
AGGCATTCTATCTCCAAGTACGTGCCCATCTTGCGGTTCAAAGTCCTGGACGCTGACCACCA
ATGCTCGCGCTTACAGTACAAGAACGTGAAACCAGATGTATGTACCCGATACCTGCTCTCGT
TTGCAGCGCAATCTCCTAGATTACTTGATGATCTAACCATTACCGCGGTATTCATCTTACAGTA
CCTTAACGCTATCTGGAACGTCATTAACTTCAAGGAGGCCGAGAAGCGCTTCGCCGACGCC
CAGAAAAAC**TAA**AACATGCCCGAAGGGTCCAAACGACACCGGGCACCCAACATTTACTTCT
ACATTCCCGAAGCAATGCCCGAGGGCGGCTATATCGGAAGACGAATTTTTAATTTCGATGCC
CGGACCCGTGGTATTTATTGATTTGGGGGTTCAGTGATGCGAAGCGGGGTCGTAAATGTCAG
ACCTGTTTCCCTAGAGGCTTGGTGTCCTGCGTGTTTTCGTTTTGGTACTATATGTCGTAGCAAT
GCAAAGCGCACGTTTAACTTTGTTCCTTTTTTGTGGG

3. Supplementary Figures

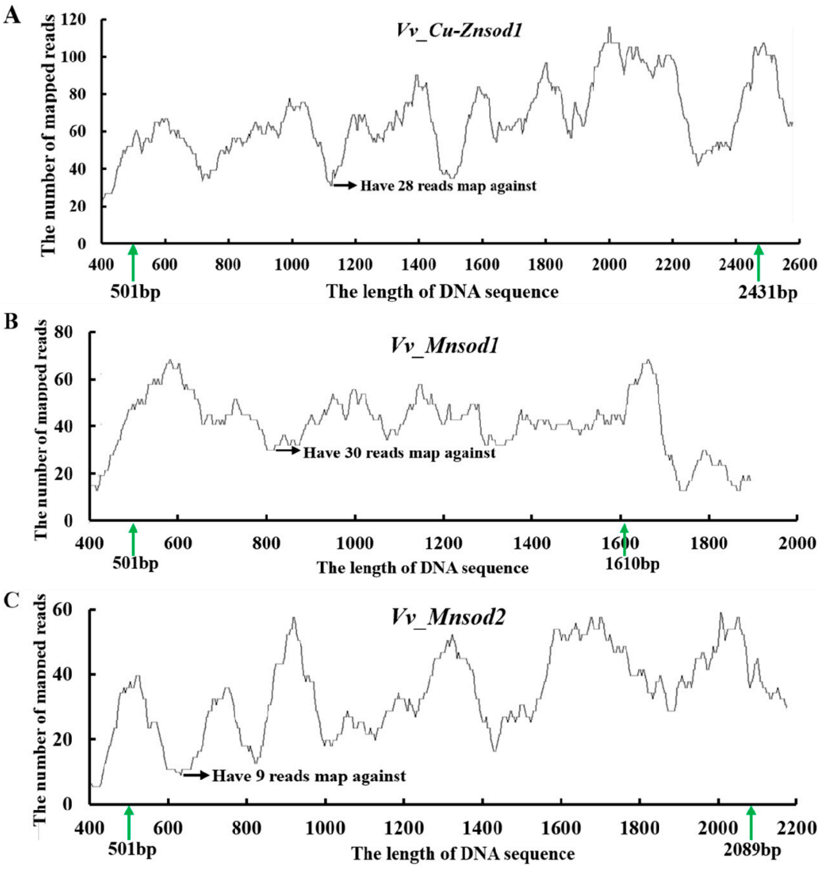

Figure S1. The mapping results of the genome sequencing reads and the *sod* sequences obtained by ZOOM software. (**A**) the genome reads mapping result of *Vv_Cu-Znsod1* gene sequence; (**B**) the genome reads mapping result of *Vv_Mnsod1* gene sequence; (**C**) the genome reads mapping result of *Vv_Mnsod2* gene sequence.

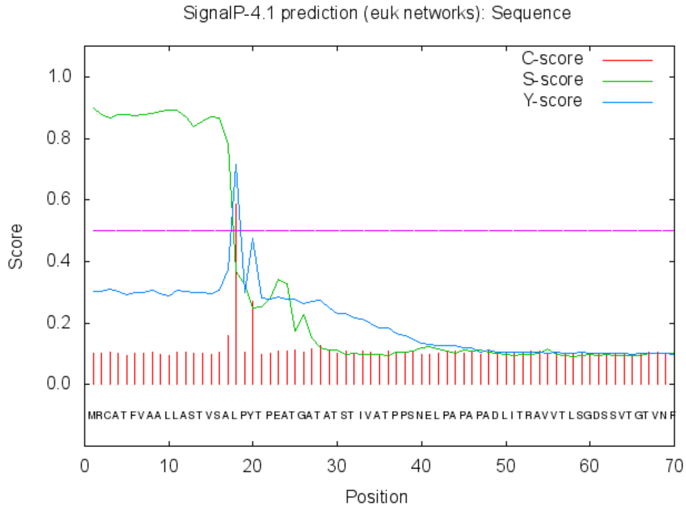

Figure S2. The signal peptide prediction result of the Vv_Cu-ZnSOD1 protein sequence. The purple line indicate the discrimination score which is used to discriminate signal peptides from non-signal peptides.

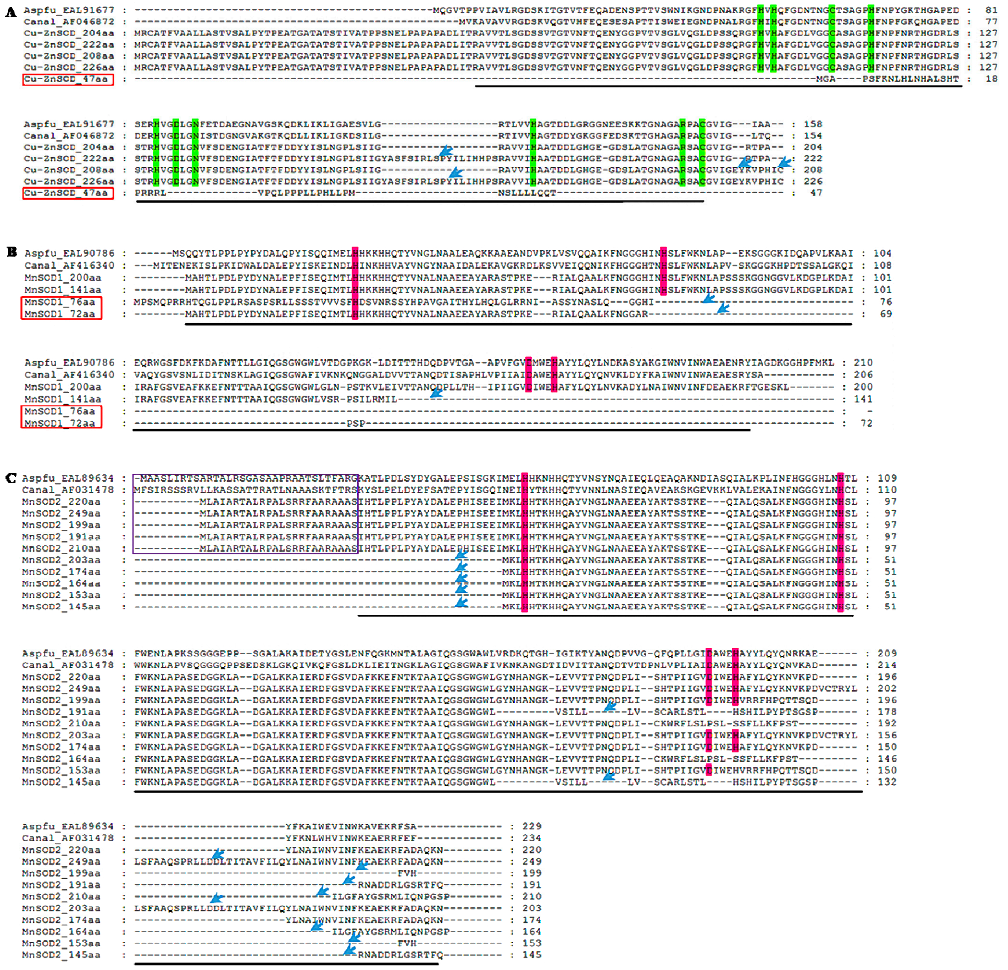

Figure S3. The lack of a SOD conserved structure domain caused by alternative splicing. Note: The sequences were downloaded from the NCBI database (http://www.ncbi.nlm.nih.gov/protein/) except for the *Volvariella volvacea* SODs. (**A**) The metal binding sites, disulfide bond, and potential glycosylation site are highlighted in green [11,12]; (**B**,**C**) The potential metal-binding sites for Mn^{2+} are shown in pink [13]. The positions of the arrows indicate the lost and gained amino acid sequences caused by alternative splicing. The transverse lines indicate the SOD conserved motif. The sequences named in the red boxes are missing large parts of the SOD motif region.

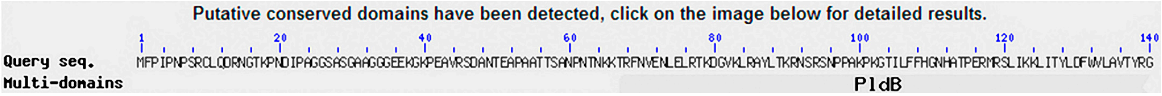

Figure S4. The structural domain prediction result of the GME5781 amino acid sequence.
